# Vehicular crash data used to rank intersections by injury crash frequency and severity

**DOI:** 10.1016/j.dib.2016.06.046

**Published:** 2016-07-01

**Authors:** Yi Liu, Zongzhi Li, Jingxian Liu, Harshingar Patel

**Affiliations:** a Wuhan University of Technology, China; b Illinois Institute of Technology, United States

## Abstract

This article contains data on research conducted in “A double standard model for allocating limited emergency medical service vehicle resources ensuring service reliability” (Liu et al., 2016) [1]. The crash counts were sorted out from comprehensive crash records of over one thousand major signalized intersections in the city of Chicago from 2004 to 2010. For each intersection, vehicular crashes were counted by crash severity levels, including fatal, injury Types A, B, and C for major, moderate, and minor injury levels, property damage only (PDO), and unknown. The crash data was further used to rank intersections by equivalent injury crash frequency. The top 200 intersections with the highest number of crash occurrences identified based on crash frequency- and severity-based scenarios are shared in this brief. The provided data would be a valuable source for research in urban traffic safety analysis and could also be utilized to examine the effectiveness of traffic safety improvement planning and programming, intersection design enhancement, incident and emergency management, and law enforcement strategies.

## Specifications Table

**Table:** 

Subject area	*Transportation engineering*
More specific subject area	*Traffic safety*
Type of data	*Figures, tables, Excel files*
How data was acquired	*Sorted out from raw crash records*
Data format	*Raw*
Experimental factors	*Intersection crashes data were extracted from raw crash records and sorted out by crash severity levels.*
Experimental features	*Intersections were ranked by crash frequency, which determined by total number of fatal and injury crashes, and crash severity, which defined by injury Type C crash equivalents*
Data source location	*Transportation Engineering Laboratory, Department of Civil, Architectural and Environmental Engineering, Illinois Institute of Technology, Chicago, IL 60616*
Data accessibility	*Data is with this article*

## Value of the data

• The shared crash data provides long-term successive counts of vehicular crashes at a large number of major signalized intersections in the city of Chicago urban street network.
• Vehicular crashes were categorized by crash severity levels, including fatal, injury Types A, B, and C, property damage only (PDO), and unknown. This is essential for the development of experiments that are in need of crash severity information.
• The shared ranking data provides top 200 intersections according to proper indicators of crash frequency and crash severity at site.
• The data sets are ponderable sources in the development of further studies in traffic safety analysis and those in need of vehicular crash count data.

## 1 Data

Data contained in this brief (spreadsheets in Supplementary data) was used to support the development of experiments that conducted by Liu et al. [1]. Vehicular crashes located inside of or near intersection are counted and categorized by crash severity levels. The data involves all recorded crashes happened in a seven-year period from year 2004 to 2010 within the city of Chicago׳s jurisdiction. In addition, the top 200 intersections are listed in the shared data according to crash frequency and crash severity levels.

## 2 Experimental design, materials and methods

### 2.1 Raw crash records

Comprehensive records of vehicular crashes occurred in the city of Chicago from 2004 to 2010 were collected and processed as the basis of the crash data shared in this article. Information documented in the raw crash records consists of crash location, year, crash severity, and crash type.

### 2.2 Intersection crash data

The shared intersection crash data was extracted and sorted out from collected raw crash records. Vehicular crashes located inside of or near an intersection were defined as intersection crashes and connected to the specific location point. Besides, when assigning crashes to a specific intersection, crash counts were classified by crash severity levels, including 1) fatal crash that involves fatality; 2) injury Types A, B, and C concerning major, moderate, and minor injury, respectively; 3) property damage only (PDO) crash with no injury; and 4) unknown that lacked key information in the raw records. Table 1 shows the temporal distribution of all intersection related crash counts in different severity levels and in total. The source file of the intersection crash data is provided in the Supplementary file 1.

### 2.3 Intersection rankings based on crash data

**Fig. 1:**
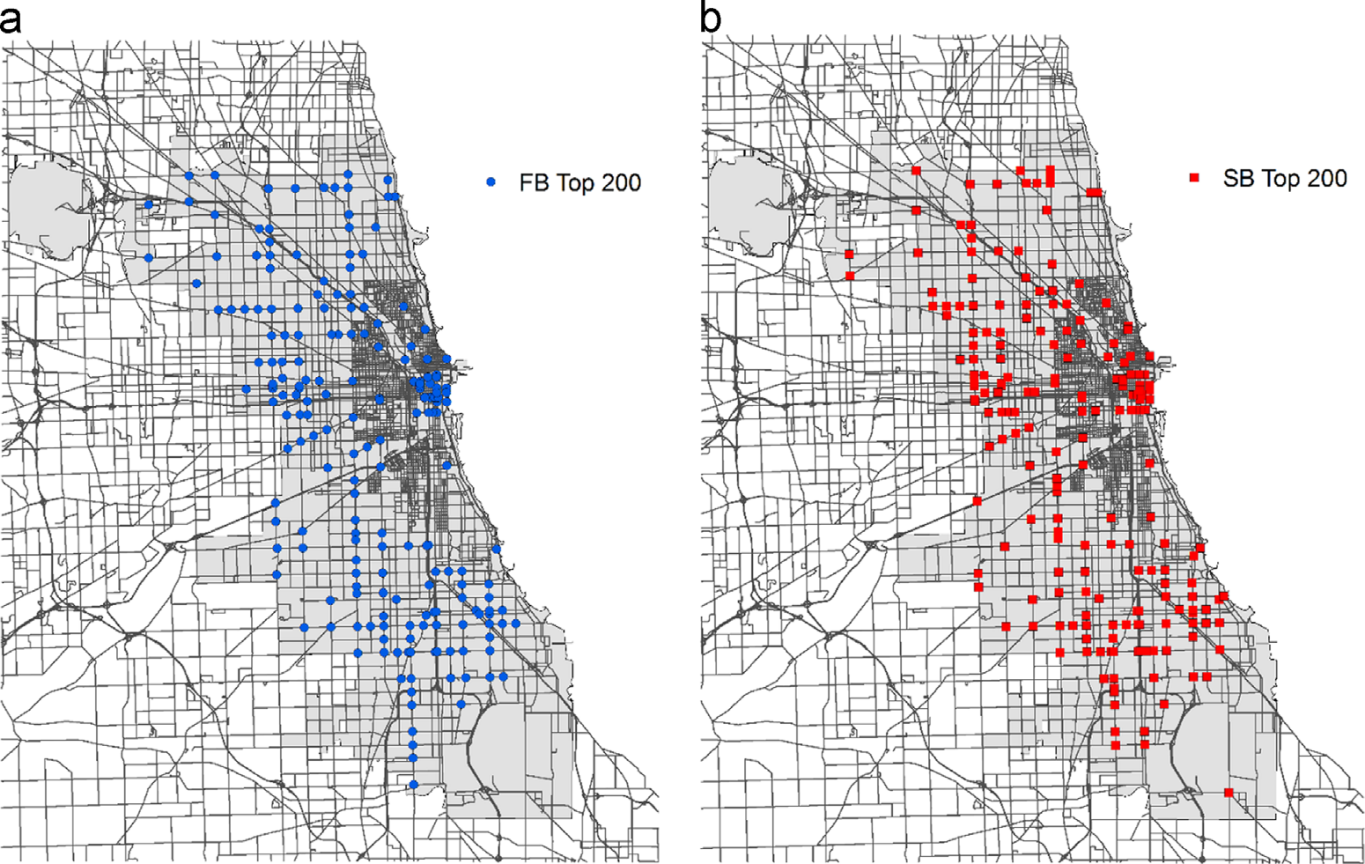
(a) Locations of top 200 intersections according to crash frequency-based (FB) ranking and (b) crash severity-based (SB) ranking in the City of Chicago׳s jurisdiction.

The intersection crash data was then used to rank intersections from the perspective of injury crash frequency and crash severity, respectively. Two traffic safety metrics were utilized for properly indicating comparable values of crash frequency and severity level at all intersections. For the frequency-based ranking, summation of numbers of fatal and all types of injury crashes was employed. While for the severity-based ranking, severity levels were unified by convert fatal and all types of injury crashes into Type B injury crash equivalents based on their estimated crash cost recommended in Highway Safety Manual (HSM) [2]. Fig. 1 illustrates locations of top 200 intersections according to crash frequency and crash severity in the City of Chicago׳s jurisdiction. Intersections were precisely positioned by using location information provided in the data sets.

The source files of ranked intersections data sets are provided in the Appendix A, Appendix A.

## Figures and Tables

**Table 1:** Distribution of intersection related crashes in different severity levels.

Crash type		2004	2005	2006	2007	2008	2009	2010
Severity level	Fatal	9	9	4	8	2	2	0
	Injury A	345	289	263	210	197	245	220
	Injury B	1418	1333	1179	900	1332	1105	1079
	Injury C	1055	855	736	636	598	841	829
	PDO	6577	5756	4949	4520	7036	10,071	10,564
	Unknown	7771	6995	7047	5729	2714	1881	1006
Total		17,175	15,237	14,178	12,003	11,879	14,145	13,698

## Footnotes

Transparency document Transparency data associated with this article can be found in the online version at doi:10.1016/j.dib.2016.06.046.

Appendix A Supplementary data associated with this article can be found in the online version at doi:10.1016/j.dib.2016.06.046.
